# Estimating the effect of realistic improvements of metformin adherence on COVID-19 mortality using targeted machine learning

**DOI:** 10.1016/j.gloepi.2024.100142

**Published:** 2024-03-30

**Authors:** Sky Qiu, Alan E. Hubbard, Juan Pablo Gutiérrez, Ganesh Pimpale, Arturo Juárez-Flores, Rakesh Ghosh, Iván de Jesús Ascencio-Montiel, Stefano M. Bertozzi

**Affiliations:** a University of California, School of Public Health, Berkeley, CA, USA; b Center for Policy, Population and Health Research, School of Medicine, Universidad Nacional Autónoma de México, Mexico City, Mexico; c University of California, Department of Mechanical Engineering, Berkeley, CA, USA; d Institute for Global Health Sciences, University of California, San Francisco, CA, USA; e Instituto Mexicano del Seguro Social, CDMX, Mexico; f University of Washington, School of Public Health, Seattle, WA, USA; g Instituto Nacional de Salud Pública, Cuernavaca, MOR, Mexico

## Abstract

**Background:**

Type 2 diabetes elevates the risk of severe outcomes in COVID-19 patients, with multiple studies reporting higher case fatality rates. Metformin is a widely used medication for glycemic management. We hypothesize that improved adherence to metformin may lower COVID-19 post-infection mortality risk in this group. Utilizing data from the Mexican Social Security Institute (IMSS), we investigate the relationship between metformin adherence and mortality following COVID-19 infection in patients with chronic metformin prescriptions.

**Methods:**

This is a retrospective cohort study consisting of 61,180 IMSS beneficiaries who received a positive polymerase chain reaction (PCR) or rapid test for SARS-CoV-2 and had at least two consecutive months of metformin prescriptions prior to the positive test. The hypothetical intervention is improved adherence to metformin, measured by proportion of days covered (PDC), with the comparison being the observed metformin adherence values. The primary outcome is all-cause mortality following COVID-19 infection. We defined the causal parameter using shift intervention, an example of modified treatment policies. We used the targeted learning framework for estimation of the target estimand.

**Findings:**

Among COVID-19 positive patients with chronic metformin prescriptions, we found that a 5% and 10% absolute increase in metformin adherence is associated with a respective 0.26% (95% CI: −0.28%, 0.79%) and 1.26% (95% CI: 0.72%, 1.80%) absolute decrease in mortality risk.

**Interpretation:**

Subject to the limitations of a real-world data study, our results indicate a causal association between improved metformin adherence and reduced COVID-19 post-infection mortality risk.

## Introduction

Multiple studies have reported higher COVID-19 case fatality rates among patients with pre-existing type 2 diabetes [[1], [2], [3], [4]]. This increased mortality has also been documented in Mexico [5], where diabetes prevalence is substantial, affecting 14% of those aged 20 years and above and reaching up to 36% among individuals aged 60 to 69 years. A substantial body of research suggests that improved adherence to glycemic control medications may reduce all-cause mortality risk (see Appendix A for a brief review) [[6], [7], [8], [9], [10], [11]]. The potential of repurposing metformin, a widely used medication for glycemic management in type 2 diabetes, as a treatment for COVID-19 has sparked recent interest due to its suggested therapeutic effects against the virus [12], including its ability to inhibit SARS-CoV-2 virus in cell cultures [13]. Because metformin is a long-term medication, poor adherence may reduce its effectiveness in glycemic control. Quantifying the degree to which improved adherence to metformin may influence COVID-19-related mortality remains a critical yet unresolved question. In this study, we evaluate the association between improved adherence to metformin and post-COVID-19 mortality by using data from the Mexican Institute of Social Security (IMSS). Metformin is the most prescribed chronic disease medication in the prescription database of IMSS, allowing us to obtain more accurate estimates of adherence. Our study population includes individuals who have health insurance coverage provided by IMSS, which consists of individuals working in the formal private sector and their families. The hypothetical intervention of interest is improved adherence to metformin, measured by proportion of days covered (PDC), with the comparison being the observed metformin adherence values. The primary outcome is all-cause mortality following COVID-19 infection. We leverage the comprehensive pharmacy prescription database provided by IMSS, the nation's largest healthcare provider. Our study spans the first three waves of the pandemic from March 2020 to December 2021.

Assessing the impacts of medication adherence using real-world data presents numerous challenges, predominantly due to the potential for confounding. The relationship between adherence and health outcomes is complex and can be confounded by a variety of patient, provider, and external factors such as age, education, disease severity and duration, comorbidities, and socioeconomic status [[14], [15], [16]]. As an example, adherent patients might have better access to healthcare resources or higher socioeconomic status, independently improving their health outcomes. Conversely, poor adherence might be associated with more severe disease or additional comorbidities, which independently lead to adverse health outcomes. Such confounding effects may exaggerate observed medication adherence benefits. On the other hand, patients with severe illness may receive stricter medication adherence recommendations, or be more motivated to adhere, leading to an observed association between improved medication adherence and negative outcomes including death.

Targeted minimum loss-based estimation (TMLE) within the targeted machine learning framework [17] has made significant advances in mitigating bias from model misspecification by enabling the use of an ensemble of state-of-the-art, flexible, machine learning algorithms (a.k.a *super learner*) to data-adaptively estimate the exposure-outcome relationship, while still delivering robust statistical inference because of its targeting step [[18], [19], [20]]. This method is particularly well-suited to big data settings, such as ours. Given the large sample size, we can aggressively reduce bias without incurring significant costs to variance, thereby yielding more precise estimates. When the exposure of interest is continuous, such as ours, the choice of causal parameter is critical as certain parameters may have less support in the data, rendering their estimates unreliable. Conventional parameters used in the context of medication adherence, such as the average treatment effect, typically attempt to measure the impact of highly improbable interventions (such as converting low-adherence individuals into high-adherence individuals rather than marginally improving adherence from an individual's baseline adherence) and necessitate artificial discretization of the continuous spectrum of medication adherence. Such oversimplifications can lead to loss of information and subsequently biased estimations. In contrast, we chose a parameter defined using shift intervention that satisfies two key conditions. Firstly, it has sufficient support in this data set, meaning there is enough variation within baseline groups to measure the targeted impact. Secondly, it provides an estimate of a realistic intervention – a small improvement on an individual's adherence. This target parameter can deliver more actionable and interpretable insights for policymakers and practitioners alike.

## Methods

### Study design and population

This is a retrospective cohort study that draws upon a population of beneficiaries of the Mexican Institute of Social Security (IMSS). We included 61,180 individuals who received a positive polymerase chain reaction (PCR) or rapid test for SARS-CoV-2 and had a history of at least two consecutive months of metformin prescriptions prior to the positive test. Data was sourced from the IMSS Epidemiological Surveillance Online Notification System (SINOLAVE) and the IMSS pharmacy prescription database, allowing for the identification of patient demographics, COVID-19 test status, deceased status, and medication prescription history. A comprehensive description of the SINOLAVE data has been published previously [21]. The three epidemiological waves are defined as: the first wave from March 29th to October 3rd, 2020; the second wave from October 4th, 2020 to May 29th, 2021; the third wave from May 30th to December 18th, 2021 [22]. The period of COVID-19 tests extended from March 2020 to October 2021, while prescription data spans from January 2018 to October 2021.

A range of potential confounders (a total of 14), both at the individual and facility level, were included in this analysis. Individual-level variables encompass age, sex, pre-existing conditions, duration on metformin, and the number of COVID-19 vaccine doses received. Facility-level variables include total population, proportion of the population with IMSS medical services affiliation, disabled population proportion, average education level, illiteracy rate, and average number of occupants per room (housing density). Only 17 patients with incomplete data on pre-existing conditions were excluded from the study. Facility-level variables contain between 3% to 12% missing data (for descriptive statistics on the distributions of metformin adherence, confounders, and COVID-19 mortality, refer to Appendix B). Missing values were imputed using respective median values, and an indicator variable was added to denote missing data for each variable with missing values.

### Medication adherence measure

We measure medication adherence [23] using proportion of days covered (PDC), calculated by dividing the number of days covered by the medication dispensed by the total duration for which an individual is prescribed that medication [24]. The Pharmacy Quality Alliance (PQA) endorses the use of PDC as a reliable metric for assessing medication adherence [25]. The medication we consider in our study is metformin, with 30 tablets per box, each tablet containing 850 mg of metformin hydrochloride, administered orally. A detailed description on the calculation of PDC and assumptions involved can be found in Appendix C.

PDC is traditionally categorized into “adherent” and “non-adherent” based on an established cutoff value [24]. A PDC value greater than or equal to 0.8 is conventionally considered sufficient for adherence and is an accepted cutoff for all class diabetes drugs as suggested by the PQA. Despite its widespread use, the justification for this cutoff value is seldom discussed in the literature. Baumgartner et al. suggested in their comprehensive review of medication adherence thresholds that these thresholds should correspond to an adherence rate above which the clinical outcome is deemed satisfactory, with the threshold varying depending on the disease, medication, and individual characteristics [26]. We concur with this recommendation, particularly when medication adherence is treated as an intervention in causal inference studies. Dichotomizing medication adherence measures based on a fixed threshold may lead to several issues. It may obscure the potential impact of differential access to medical resources on medication adherence, leading to less interpretable effects due to such inequalities. Moreover, dichotomization can lead to positivity violation–a lack of data support for individuals with certain characteristics–that may result in biased estimates, large variances, and uninterpretable estimates [27]. Additionally, if we adopt 0.8 as the cutoff for PDC, we lose the ability to distinguish the impact of 0.7 adherence on a clinical outcome from that of 0.1 adherence. Thus, crucial information embedded in the continuous nature of medication adherence may be lost due to dichotomization.

To address these concerns, we propose defining a causal parameter for medication adherence using *shift intervention* [[28], [29], [30]], an innovative approach for quantifying causal effects of interventions on continuous variables (treatments or exposures). Specifically, our causal parameter is defined as the difference between the observed mortality risk and the expected mortality risk in a hypothetical world wherein everyone's medication adherence is increased by a small amount. The shift intervention approach obviates the need for arbitrary dichotomization of medication adherence measures and allows for the formulation of more flexible and feasible causal parameters that are less susceptible to positivity violations [30].

### Structural causal model, causal parameter, and identification

The principal objective of this study is to assess the causal effect of metformin adherence on COVID-19-related mortality, adjusting for confounding factors. For this purpose, we employ a nonparametric structural equation model (NPSEM) [19] to model the data-generating process, with W denoting confounders, A representing the adherence measure (PDC), and Y indicating deceased status: $W=f_{W}\left(U_{W}\right),A=f_{A}\left(W,U_{A}\right),Y=f_{Y}\left(W,A,U_{Y}\right)$, where $U_{W}$, $U_{A}$, and $U_{Y}$ are variables for unobserved exogenous errors. The functional forms of $f_{W}$, $f_{A}$, and $f_{Y}$ are unspecified, hence nonparametric. We observe data for n patients. The observed data for the *i*-th patient is given by $O_{i}=\left(W_{i},A_{i},Y_{i}\right)$, which is drawn from the unknown true data-generating distribution, $P_{0}$, of the target population. We assume that $O_{1},O_{2},\ldots ,O_{n}$ are independent and identically distributed.

Shift intervention is one example of a modified treatment policy (MTP), in which the hypothetical intervention is characterized by a constant shift in the distribution of the observed treatment [29,31]. Shift intervention can be used to define causal parameters when the treatment is continuous such as medication adherence in our study. Consider an additive shift intervention that adds *δ >* 0 to everyone's medication adherence. We are interested in the causal effect of such *δ*-shift of adherence on COVID-19 mortality. We define our causal parameter as the decrease in the expected COVID-19 mortality after the *δ*-shift of adherence. Formally, suppose the distribution of metformin adherence Aconditioned on patient characteristics W=whas support on the interval $\left[l\left(w\right),u\left(w\right)\right]$, we define the treatment rule d based on a *δ*-shift of observed treatment for a patient with observed metformin adherence A=a and characteristics W=w as $d\left(a,w;\delta \right)=a+\delta$ if $a\leq u\left(w\right)-\delta$ and $d\left(a,w;\delta \right)=a$ if $a>u\left(w\right)-\delta$. In other words, we only shift the metformin adherence of a patient if there is still sufficient data support under such shift (we cannot improve adherence to >100%). Following the potential outcomes framework [32], our target parameter is $\Psi \left(P\right)=E_{P}\left(Y\right)-E_{P}\left(Y^{d}\right)$, where $Y^{d}$ is the potential outcome of deceased status Y under the treatment rule d characterized by the *δ*-shift intervention.

We made two key assumptions for the identification of the causal parameter. Firstly, the randomization assumption presumes that there are no unmeasured variables affecting both the treatment and the outcome. Secondly, the positivity assumption states that the shifted treatment for a patient with specific characteristics still lies within the support of the observed treatment given those characteristics. In this context, the randomization assumption infers the absence of unmeasured variables affecting both metformin adherence and COVID-19 mortality. For example, disease severity of diabetes could potentially confound this relationship. The randomization assumption is made more plausible by adjusting for variables that might reflect disease severity, such as age, total number of metformin prescriptions, etc. However, the validity of this assumption cannot be assured, primarily due to the absence of data on critical markers like HbA1C values in patients. For the positivity assumption, in the context of our study, it suggests that the shifted level of metformin adherence for a patient, given their specific characteristics (like age, pre-existing conditions, etc.), should still lie within the range of observed adherence levels for patients with similar characteristics. Intuitively, this assumption ensures that our comparisons are “like with like” and that our intervention levels are not extrapolated beyond the data. However, it is hard to empirically validate this assumption as it requires knowledge about the full intervention distribution for everyone, which is unobservable in practice. To make this assumption more plausible, we chose two shifts of PDC value, 0.05 and 0.1 increase of metformin adherence on the zero to one scale of percent adherence, that are within the realm of what is practically achievable based on our data support and likely to be clinically relevant. The choice of the two shift threshold values is determined such that the estimated ratio between the conditional density of the adherence under the counterfactual *δ*-shift and the conditional density of the observed adherence (conditioned on patient characteristics) remains below 10. With these two assumptions, we can express our causal parameter Ψ in terms of the observed data distribution $P_{0}$ to construct a causal estimand, given by $\Psi \left(P_{0}\right)=E_{0}\left(Y\right)-E_{0}E_{0}\left(Y|A=d\left(A,W;\delta \right),W\right)$. Thus, positive values of $\Psi \left(P_{0}\right)$ indicate reduced mortality risk under the shift intervention.

### Estimation and inference

For the estimation of the causal parameter, we employed the targeted minimum loss-based estimation (TMLE), which provides an asymptotically linear estimator with an optimal bias-variance trade-off, specifically tailored towards the target parameter of interest [19,20]. The estimator constructed under the TMLE framework is double-robust, meaning that if either the outcome regression model, ${\bar{Q}}_{0}\left(A,W\right)=E_{0}\left(Y|A,W\right)$, or the conditional density of medication adherence, $g_{0}\left(A|W\right)=p_{0}\left(A|W\right)$, is correctly specified, the causal effect estimate would be unbiased. TMLE yields a substitution estimator, which ensures that the estimated causal effect remains within its possible range [20].

The estimation process involves two steps. Firstly, an initial substitution estimator was constructed, with the empirical mean of the COVID-19 mortality, i.e., $\bar{Y}=\frac{1}{n}\sum_{i}Y_{i}$, used as an estimator for $E_{0}\left(Y\right)$. The outcome regression ${\bar{Q}}_{0}\left(A,W\right)$ was estimated using super learner, a cross-validated ensemble machine learning algorithm, with metformin adherence A and patient characteristics W as covariates and COVID-19 mortality Y as the outcome. For the marginal distribution of W, we use its empirical distribution as an estimator. Secondly, the estimated ${\bar{Q}}_{n}\left(A,W\right)$ from the first step was fluctuated using a parametric submodel, resulting in an updated ${\bar{Q}}_{n}^{*}\left(A,W\right)$. Thus, the updated estimator for $E_{0}E_{0}\left(Y|A=d\left(A,W;\delta \right),W\right)$ is given by $\frac{1}{n}\sum_{i}{\bar{Q}}_{n}^{*}\left(d\left(A_{i},W_{i};\delta \right),W_{i}\right)$. The resulting estimator is asymptotically linear, permitting the construction of a Wald-type 95% confidence interval around the point estimate. Details of the estimation steps can be found in Appendix B. The learners and their hyperparameters used in the super learner libraries for both ${\bar{Q}}_{0}$ and $g_{0}$ are available in Appendix C.

It is worth considering that the effect of metformin adherence on COVID-19 mortality may differ across the various waves of the pandemic. Prior research using data from the IMSS suggests that the relationship between risk factors and COVID-19 mortality declined over successive waves [22]. Moreover, the evolving nature of governmental interventions in response to the pandemic [33] may further modulate the impact of metformin adherence on COVID-19 mortality. Therefore, we also conduct a stratified analysis to examine the causal effect estimate across different COVID-19 waves. To verify our findings' robustness, we conduct a sensitivity analysis using the COVID-19 test result as a negative control outcome. We hypothesized that while metformin adherence shares common confounders with the COVID-19 test result, there should not be a causal relationship between them. Consequently, a null effect of metformin adherence on the COVID-19 test result estimated using the same statistical analysis approach would further corroborate the robustness of the methodology we employed in our study.

## Results

From the IMSS prescription database, we retrieved a total of 5,959,306 metformin prescriptions associated with 433,779 patients. Among these patients, 90,812 had at least one documented positive result from a polymerase chain reaction (PCR) or rapid test for SARS-CoV-2. After eliminating expired prescriptions that were never filled and patients with a single metformin prescription, the final data set for analysis comprised 61,180 individuals. Of those individuals, classified by their most recent positive test, 21,715 were in wave 1, 28,425 were in wave 2, and 11,040 were in wave 3. The average PDC across the waves was 0.81 (sd = 0*.*13), 0.81 (sd = 0*.*13), and 0.82 (sd = 0*.*13), respectively. Corresponding mortality rates were 31.8%, 35.9%, and 35.8%. Details of patient characteristics are provided in Appendix B.

**Fig. 1:**
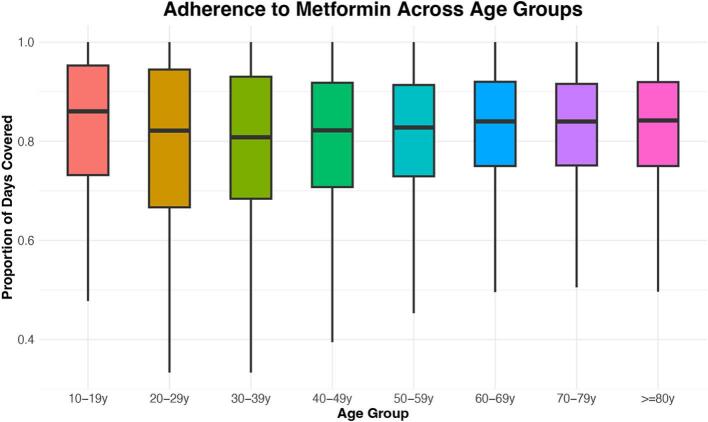
Side-by-side box plot for the distribution of PDC across age groups.

Fig. 1 shows the distribution of metformin adherence across different age demographics. The median proportion of days covered (PDC) initially descends among patients aged 10–39 years, ascends to a peak among those in the 60–69 years age bracket, and then plateaus for individuals over 70 years. The interquartile range (IQR) generally narrows with advancing age, except for the youngest cohort. The youngest patient group (10–19 years old) exhibits the highest median PDC. We hypothesize that this may be associated with the high proportion of type 1 diabetics among the 10–19 diabetic population, requiring greater adherence to avoid life-threatening hypo- or hyper-glycemia, as well as oversight from parents or guardians helping to ensure that medications are taken punctually. Conversely, lower adherence levels observed among young adults could stem from lower diabetes severity in a population where the majority of diabetes is now type 2.

**Fig. 2:**
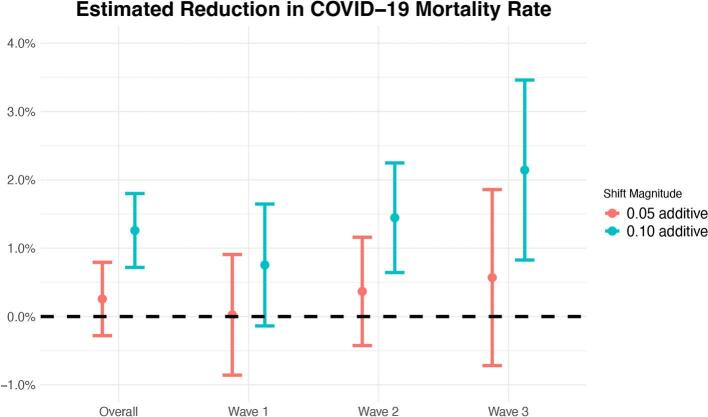
Estimated reduction in COVID-19 post-infection mortality rate overall and stratified by wave under 0.05 and 0.10 additive shifts in metformin adherence, adjusting for confounders. The error bars are 95% confidence intervals obtained using the efficient influence function approach (see Appendix C).

In Fig. 2, we present the estimated impact of two interventions - additive shifts of 0.05 and 0.1 in metformin adherence - on the reduction of COVID-19 mortality rates across all waves and within each individual wave. Under the randomization and positivity assumptions outlined in the Methods section, we can interpret these effect estimates causally. A 0.1-additive shift in metformin adherence would lead to an estimated reduction in COVID-19 mortality of 1.26% (95% CI: 0.72%, 1.80%) for all COVID-19 waves combined. The estimated reductions in COVID-19 mortality are 0.75% (95% CI: −0.14%, 1.65%), 1.45% (95% CI: 0.64%, 2.25%), and 2.14% (95% CI: 0.83%, 3.46%) respectively for the first, second, and third COVID-19 wave. From the first to the third wave, the estimated decrease in COVID-19 mortality progressively increases.

**Fig. 3:**
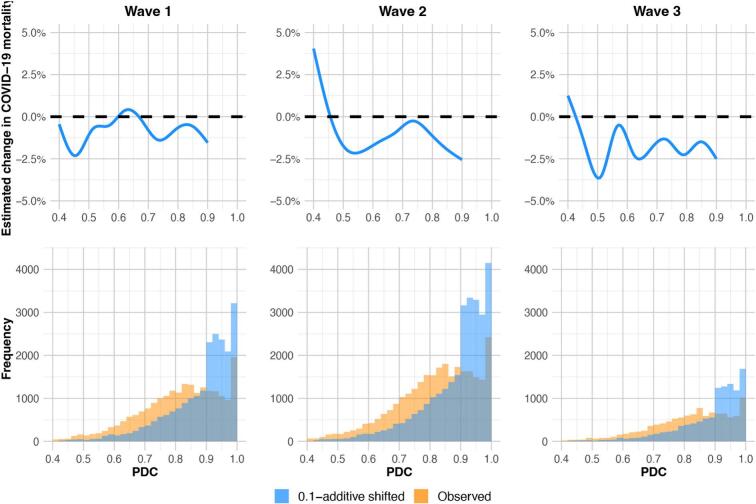
Super learner-based substitution estimates of the change in COVID-19 mortality and the empirical distribution of metformin adherence before and after the 0.1-additive shift. The curves in the top three plots are fitted using generalized additive models on the super learner predicted change in COVID-19 mortality risk after the 0.1-additive shift on every subject for wave 1, 2, and 3 respectively. The bottom three histograms are the empirical distributions of metformin adherence (measured using PDC) before and after the 0.1-additive shift for wave 1, 2, and 3 respectively.

To facilitate the visualization of the impact of improved metformin adherence across a range of baseline adherence values and add more transparency to the super learner ensemble model predictions, we predicted the change in COVID-19 mortality risk before and after the shift for every subject using the fitted super learner. We estimated the COVID-19 mortality risk before the shift by taking the empirical proportion. The top three plots in Fig. 3 show smooth curves fitted using generalized additive models on the super learner predicted change in mortality risk after the 0.1-additive shift for each subject. The smooth curves predominantly reside below zero, suggesting an overall positive effect of improved metformin adherence on the reduction of COVID-19 mortality risk. Note that individuals with baseline PDC above 0.9 were not shifted, therefore there is no change in the estimated mortality. The histograms in the bottom panel depict the distributions of baseline and 0.1-shifted adherence values for each COVID-19 wave.

## Discussion

In our study, we found better metformin adherence diminishes mortality risk after COVID-19 infection, as highlighted by our shift intervention-defined causal target parameter. For context, envision a patient on metformin for 14 months (the median span for our study group). An adherence improvement equivalent to roughly one and a half months is associated with a 1.26% (95% CI: 0.72%, 1.80%) absolute reduction in COVID-19 post-infection mortality risk. Our findings emphasize the potential of shift intervention techniques in advancing causal inference studies when the exposure variable is continuous in nature. Compared to conventional methodologies that categorize adherence levels in a binary fashion, shift interventions enable interventions to be contingent upon the naturally observed adherence values of individual subjects. This approach obviates the necessity for arbitrarily defining a threshold value and concurrently mitigates the violation of the positivity assumption. MTPs have garnered increasing attention within the causal inference community in recent years [30,[34], [35], [36], [37]]. Our investigation is among the pioneering efforts to operationalize this approach. During the statistical estimation phase, we employed the targeted minimum loss-based estimation (TMLE) framework. In contrast to parametric regression techniques (e.g., logistic regression) frequently utilized in existing research, TMLE utilizes an ensemble of state-of-the-art machine learning algorithms to capture the intricacies of the underlying data distribution, while also producing an estimator conducive to robust statistical inference.

### Practical implications

Our results suggest that a 0.1 increase in metformin adherence reduces COVID-19 mortality. A smaller shift of 0.05 produces a similar pattern. A noticeable trend across COVID-19 waves in Fig. 2 suggests that the temporal heterogeneity of the effect may be influenced by factors such as COVID-19 vaccination rates, changes in the probability of prior infection, and/or changes in the circulating mix of viral variants. Wave 1, spanning from March to October 2020, occurred before the availability of COVID-19 vaccinations in our data. COVID-19 vaccinations might augment the impact of metformin adherence. To explore this hypothesis, we conducted additional analyses comparing effect estimates between unvaccinated individuals and those who received at least one COVID-19 vaccine dose. Under a 0.05 shift, the estimated effects were 0.24% (95% CI: −0.31%, 0.79%) for unvaccinated individuals and 0.95% (95% CI: −1.46%, 3.36%) for vaccinated ones. Under a 0.1 shift, the corresponding estimates were 1.08% (95% CI: 0.52%, 1.63%) and 2.08% (95% CI: −0.78%, 4.94%). Though the differences in the estimated effects between the vaccinated and unvaccinated individuals are not significant due to a limited vaccinated sample size, point estimates are noticeably higher among vaccinated individuals, suggesting that it would be interesting to explore this further with additional data. In addition to vaccination efforts, previous study has reported a notable decline in diabetes prevalence among individuals who tested positive for COVID-19 [38]. They also pointed out that as the disease progresses, healthcare management of severe cases gets better. The beneficial effect of metformin on COVID-19 mortality may be overwhelmed by the high case fatality rate in the first wave. As the disease progresses, the improvement in severe case management, in conjunction with changing epidemiological profiles of infected individuals, public health policies, healthcare system capacities, and the dynamic nature of the virus, could make the effect of metformin adherence on COVID-19 mortality risk more pronounced and hence explain the time-varying effect we observe.

We are also aware of recent work exploring the possibility that metformin may have a direct effect on COVID-19 severity, independent of its effect at improving glycemic control in diabetic patients [39]. Clinical trials have been conducted to evaluate this effect [[40], [41], [42]]. Evidence from clinical trials does not show conclusive evidence suggesting the beneficial effect of metformin on COVID-19 outcomes. However, the COVID-OUT trial found evidence suggesting that metformin may reduce long COVID incidence. Our IMSS colleagues are not aware of any prescription of metformin as a COVID-19 prophylactic agent in the IMSS, and thus we believe that our sample is not affected by such off-label use. However, metformin is increasingly prescribed for pre-diabetic patients as evidence suggests that it help to forestall the development of type 2 diabetes [43,44]. If subsequent analyses were able to access outpatient visit data as well as prescription data, it would be possible to do a sub-analysis of patients with pre-diabetes, with and without metformin prescriptions. A small proportion of the metformin prescriptions were to patients who self-reported either chronic liver disease or chronic renal disease. In light of these conditions being relative contraindications for use of metformin, it would be appropriate to review a sample of those cases to see if it would be appropriate to institute protections to ensure that metformin is only prescribed for such patients following a careful assessment of the potential risks and benefits.

### PDC as a measure of medication adherence

One limitation of our study is the potential overestimation of true medication adherence by the PDC measure [45]. In calculating PDC, we postulated that any remaining medications from the current month would be carried over to the subsequent month. However, in actuality, medications may be misplaced, or patients may not consume all prescribed pills. Although PDC overestimation may introduce bias into our findings, we contend that our methodology remains more robust in comparison to conventional techniques that dichotomize adherence. The PDC values we calculated may result in conservative effect estimates. Therefore, we expect the true causal relationship to be larger if assumptions we made in calculating PDC is violated. We refer interested readers to Appendix C for a more detailed discussion on the calculation of PDC.

In future studies, one may also consider a multifaceted approach for measuring medication adherence along with PDC. For example, researchers could consider text messages/phone interviews asking whether patients are taking their medications on time. Researchers could also identify potential biomarkers that are associated with adherence. As an example, in heart failure, clinicians often examine the biomarker N-terminal pro-B-type natriuretic peptide (NT-proBNP) as a guide to assess disease management [46]. We advocate more research on biomarkers that are associated with disease management to be used in companion with PDC derived from pharmacy refill records to get a better estimate of patients' medication taking behavior.

### Effect of adherence on testing positive for COVID-19

We have no reason to believe that adherence affects the probability that a patient with respiratory symptoms has COVID, thus, as a robustness check we performed a sensitivity analysis using COVID-19 test result as a negative control outcome, examining the impact of adherence on the likelihood of obtaining a positive COVID-19 test outcome using a similar shift intervention strategy. The estimated effects for wave one, two, three are 0.32% (95% CI: −0.40%, 1.04%), −0.33% (95% CI: −0.79%, 0.12%), and − 0.35% (95% CI: −0.61%, 0.10%) respectively. The estimated null effects provide an additional layer of evidence that the method we use is robust.

## Conclusion

In conclusion, our study demonstrated that improved metformin adherence among individuals with diabetes is associated with a reduction in COVID-19 mortality, as evidenced by the causal target parameter estimates derived using shift intervention, although it is not clear what the mechanism is for this effect. Explanations include more adherent patients have better controlled diabetes or metformin directly reduces the probability of severe disease. The utilization of the shift intervention target parameter and the targeted minimum loss-based estimation (TMLE) framework facilitated a robust analysis, enabling us to assess the average effect of metformin adherence on COVID-19 mortality without relying on arbitrary threshold values or binary categorization. Although our study employed the proportion of days covered (PDC) as a measure of medication adherence, which has some limitations, our methodology remains more robust compared to conventional techniques. We encourage further research to corroborate these findings and explore additional potential confounders, particularly the level of patients' glycemic control and their adherence to other medications and health practices, ultimately enhancing our understanding of the relationship between metformin adherence and COVID-19 mortality, as well as the potential implications for public health policies and healthcare system management.

## Funding

This study is supported by C3.ai Digital Transformation Institute and Bill & Melinda Gates Foundation (OPP1165144).

## CRediT authorship contribution statement

**Sky Qiu:** Writing – review & editing, Writing – original draft, Visualization, Software, Methodology, Investigation, Formal analysis, Data curation, Conceptualization. **Alan E. Hubbard:** Writing – review & editing, Supervision, Investigation, Funding acquisition, Conceptualization. **Juan Pablo Gutiérrez:** Writing – review & editing, Supervision, Investigation, Funding acquisition, Conceptualization. **Ganesh Pimpale:** Data curation. **Arturo Juárez-Flores:** Resources, Data curation. **Rakesh Ghosh:** Writing – review & editing, Data curation. **Iván de Jesús Ascencio-Montiel:** Data curation. **Stefano M. Bertozzi:** Writing – review & editing, Supervision, Investigation, Funding acquisition, Conceptualization.

## Declaration of competing interest

The authors declare that they have no known competing financial interests or personal relationships that could have appeared to influence the work reported in this paper.

## Data Availability

Due to the confidential nature of the patient records used in this study and strict access controls, we are unable to share the data.

## Appendix A Supplementary data

Supplementary material Literature review, summarizing table for patient characteristics, details on methods, and additional figures. Supplementary material
