# Supplementary material for: Estimating the effect of realistic improvements of metformin adherence on COVID-19 mortality using targeted machine learning
Source: Glob Epidemiol. 2024 Mar 30;7:100142. doi: 10.1016/j.gloepi.2024.100142 (PMC10999684; doi:10.1016/j.gloepi.2024.100142)
Supplement: Supplementary material — Literature review, summarizing table for patient characteristics, details on methods, and additional figures. [file mmc1.pdf]

Appendix A: Literature review

Existing studies on adherence to glycemic control medications and mortality mostly focus on all-cause mortality. In a cohort of 11,532 diabetes patients, Ho et al. [6] found that non-adherence to oral hypoglycemics, antihypertensives, and statin medications was associated with increased all-cause mortality (adjusted odds ratio: 1.58, 95% CI: 1.38-1.81). Similar findings were reported by Hong and Kang [7] (adjusted odds ratio: 1.40, 95% CI: 1.01-1.95). In a 5-year survival analysis of a longitudinal cohort of 629,563 veterans with type 2 diabetes, Egede et al. [8] reported that the increase in mortality risks associated with non-adherence is highest in Hispanic veterans (adjusted hazard ratio: 12.65; 95% CI: 11.10-14.43). In another 5-year survival analysis of 159,032 veterans diagnosed with uncomplicated diabetes, Gatwood et al. [9] found veterans with $< 20\%$ adherence to oral anti-diabetic medications have higher mortality risk (hazard ratio: 1.33; 95% CI: 1.17-1.51). Kim et al. [10] also found the largest association in patients $< 20\%$ adherence (adjusted hazard ratio: 1.45, 95% CI: 1.36-1.54). In addition, they also found those with PDC $< 20\%$ had increased risk for CVD-related hospital admission. In a nested case-control study, Simard et al. [11] found decreased mortality risks after long-term adherence to metformin (4-6 years of adherence risk ratio: 0.84, 95% CI: 0.71-0.98; ≥ 6 years of adherence risk ratio: 0.69, 95% CI: 0.57-0.85).

Appendix B: Patient characteristics

Table 1 shows descriptive summary statistics on the distribution of metformin adherence, confounders, and COVID-19 mortality for each COVID-19 wave.

Table 1: Descriptive statistics on the distribution of metformin adherence, confounders, and COVID-19 post-infection mortality by wave. For continuous variables (marked using *) and proportion variables (marked using †), we show the mean with standard deviation in parentheses. For binary and categorical variables, we show the count with the corresponding proportion in parentheses.

	**Wave 1**	**Wave 2**	**Wave 3**
n	21715	28425	11040
**Variable**	**Mean/count (SD/proportion)**		
Sex = female	11439 (52.7)	14776 (52.0)	5958 (54.0)
Age*	58.90 (13.12)	60.09 (13.17)	60.64 (13.79)
Indigenous = yes	63 (0.3)	76 (0.3)	29 (0.3)
IMSS delegation			
Aguascalientes	383 (1.8)	483 (1.7)	12 (0.1)
Baja California	139 (0.6)	787 (2.8)	141 (1.3)
Baja California Sur	301 (1.4)	250 (0.9)	151 (1.4)
Campeche	124 (0.6)	42 (0.1)	212 (1.9)
Chiapas	23 (0.1)	34 (0.1)	175 (1.6)
Chihuahua	612 (2.8)	633 (2.2)	239 (2.2)
Coahuila	1368 (6.3)	1285 (4.5)	373 (3.4)
Colima	299 (1.4)	173 (0.6)	187 (1.7)
DF 1 Noroeste	351 (1.6)	656 (2.3)	283 (2.6)
DF 2 Noreste	732 (3.4)	1339 (4.7)	393 (3.6)
DF 3 Suroeste	624 (2.9)	1241 (4.4)	175 (1.6)

Continued on next page

Table 1 Continued from previous page

	Wave 1	Wave 2	Wave 3
DF 4 Sureste	861 (4.0)	1792 (6.3)	324 (2.9)
Durango	533 (2.5)	528 (1.9)	113 (1.0)
Guanajuato	1218 (5.6)	2134 (7.5)	332 (3.0)
Guerrero	211 (1.0)	285 (1.0)	86 (0.8)
Hidalgo	521 (2.4)	536 (1.9)	235 (2.1)
Jalisco	2116 (9.7)	2678 (9.4)	914 (8.3)
Mexico Oriente	1373 (6.3)	2562 (9.0)	958 (8.7)
Mexico Poniente	792 (3.6)	1090 (3.8)	160 (1.4)
Michoacan	382 (1.8)	438 (1.5)	250 (2.3)
Morelos	200 (0.9)	454 (1.6)	230 (2.1)
Nayarit	80 (0.4)	108 (0.4)	77 (0.7)
Nuevo Leon	2657 (12.2)	3351 (11.8)	1393 (12.6)
Oaxaca	135 (0.6)	123 (0.4)	117 (1.1)
Puebla	801 (3.7)	1110 (3.9)	587 (5.3)
Queretaro	116 (0.5)	461 (1.6)	83 (0.8)
Quintana Roo	379 (1.7)	196 (0.7)	243 (2.2)
San Luis Potosi	545 (2.5)	497 (1.7)	201 (1.8)
Sinaloa	467 (2.2)	286 (1.0)	344 (3.1)
Sonora	662 (3.0)	668 (2.4)	166 (1.5)
Tabasco	39 (0.2)	39 (0.1)	116 (1.1)
Tamaulipas	93 (0.4)	154 (0.5)	168 (1.5)
Tlaxcala	146 (0.7)	145 (0.5)	37 (0.3)
Veracruz Norte	869 (4.0)	567 (2.0)	447 (4.0)
Veracruz Sur	585 (2.7)	371 (1.3)	339 (3.1)

Continued on next page

Table 1 Continued from previous page

	**Wave 1**	**Wave 2**	**Wave 3**
Yucatan	527 (2.4)	256 (0.9)	554 (5.0)
Zacatecas	451 (2.1)	673 (2.4)	225 (2.0)
Average school grade*	11.75 (2.14)	11.72 (2.31)	11.49 (2.54)
Disabled population†	0.05 (0.02)	0.05 (0.02)	0.05 (0.02)
IMSS insured population†	0.55 (0.12)	0.54 (0.12)	0.54 (0.13)
Illiterate population†	0.01 (0.01)	0.01 (0.01)	0.01 (0.01)
Mean occupant per room*	0.72 (0.18)	0.72 (0.19)	0.73 (0.21)
Preexisting conditions			
COPD	937 (4.3)	1066 (3.8)	410 (3.7)
Diabetes	15182 (69.9)	18497 (65.1)	7064 (64.0)
Asthma	555 (2.6)	610 (2.1)	254 (2.3)
Immunosuppression	430 (2.0)	469 (1.6)	174 (1.6)
Obesity	5659 (26.1)	6264 (22.0)	1979 (17.9)
HIV	56 (0.3)	109 (0.4)	37 (0.3)
Hypertension	12027 (55.4)	15057 (53.0)	5761 (52.2)
Cardiovascular Disease	996 (4.6)	1158 (4.1)	496 (4.5)
Chronic Liver Disease	71 (0.3)	95 (0.3)	32 (0.3)
Hemolytic Anemia	20 (0.1)	22 (0.1)	11 (0.1)
Neurological Disease	50 (0.2)	68 (0.2)	37 (0.3)
Tuberculosis	14 (0.1)	23 (0.1)	11 (0.1)
Cancer	131 (0.6)	150 (0.5)	91 (0.8)
Renal Disease	857 (3.9)	1060 (3.7)	579 (5.2)
COVID-19 vaccine doses			
0	21715 (100.0)	28409 (99.9)	8056 (73.0)

Continued on next page

Table 1 Continued from previous page

	**Wave 1**	**Wave 2**	**Wave 3**
1	0 (0.0)	16 (0.1)	1255 (11.4)
2	0 (0.0)	0 (0.0)	1729 (15.7)
Smoker = yes	1459 (6.7)	1885 (6.6)	679 (6.2)
Months on metformin*	12.99 (8.03)	14.46 (9.16)	15.51 (10.85)
History of insulin use = yes	248 (1.1)	324 (1.1)	130 (1.2)
Proportion of days covered†	0.81 (0.13)	0.81 (0.13)	0.82 (0.13)
Death = yes	6902 (31.8)	10216 (35.9)	3956 (35.8)

Appendix C: Details on methodology

Metformin adherence measure

In our study, we use proportion of days covered (PDC) as the measure for metformin adherence. We now detail the calculation of PDC and assumptions made.

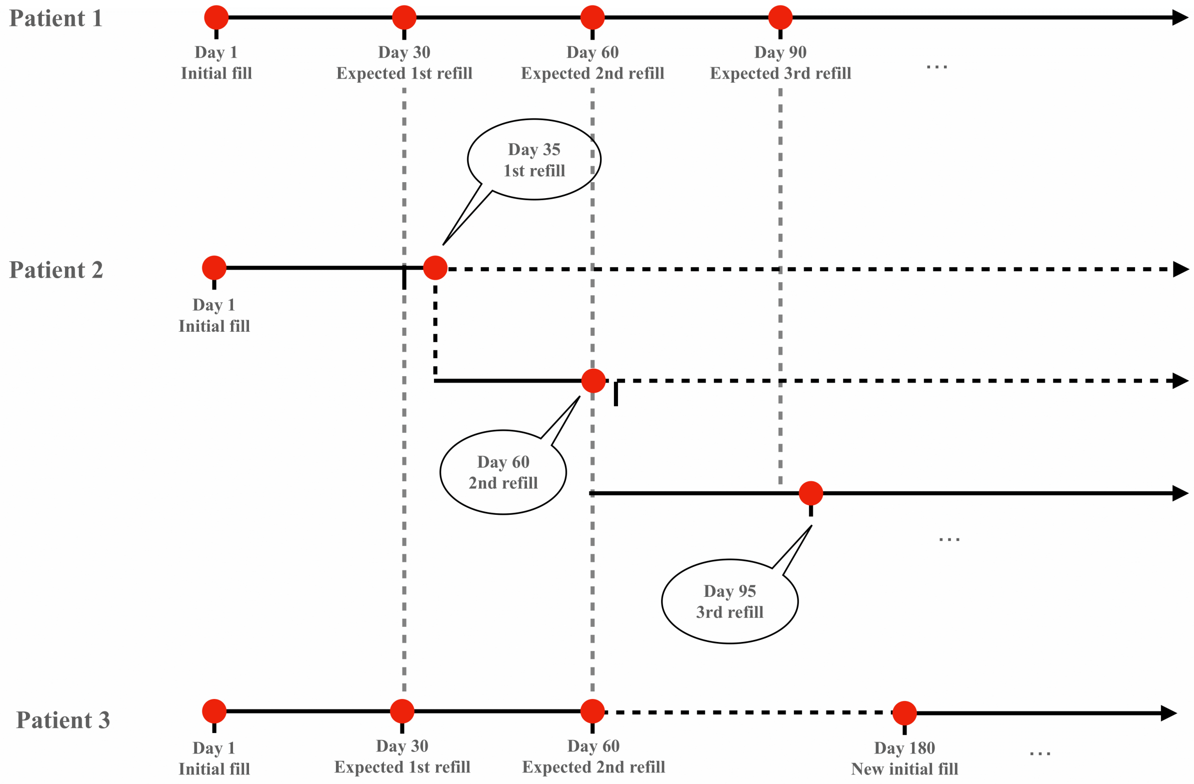

Figure 4: Examples of prescription refill patterns.

Figure 4 provides three examples of metformin prescription refill patterns, with the red dots indicating the dates when medications were collected by patients. We use these examples to demonstrate the calculation for the PDC measure. The first example showcases a patient who consistently refilled prescriptions on time, resulting in a PDC of 1. In the second example, the patient's first refill was delayed by five days, and the second refill was early by five days, while the third refill was on time. Consequently, there were five days

without medication during the first interval. However, due to an excess of medication from the early second refill, there were no days without medication in the second and third intervals. Therefore, the PDC for the second individual is 90 days/95 days ≈ 0.95. For the third patient, a gap in the prescription record arises between day 60 and day 180 where no prescription is found. In the calculation of the PDC, we opt to exclude any gap exceeding 60 days. We anticipate that this approach could lead to a conservative estimation of the intervention's impact, potentially understating the real influence of medication adherence on health outcomes for the following two reasons. First, disregarding gaps over 60 days, we may inadvertently discount periods of non-adherence that could considerably affect health outcomes. Specifically, frequent gaps may point to inconsistent medication use, potentially compromising the condition's optimal control. This inconsistency could subtly diminish the estimated treatment effect, making the intervention seem less beneficial than it may truly be. Second, these extended gaps could reflect clinical recommendations for temporary treatment cessation due to adverse reactions, disease progression, or an alternate treatment introduction. These clinical judgments carry profound implications for patient health outcomes and perceived medication effectiveness. In particular, if these periods of clinician-advised treatment discontinuation coincide with poorer health outcomes, excluding them from the PDC calculation could cause the association between medication adherence and outcomes to appear weaker than it truly is. Therefore, the PDC of the third individual is evaluated as 1 despite the presence of this extended gap. While our methodology might present a somewhat understated picture of the intervention's effect, it should be noted that this underestimation, though potentially biasing our results towards the null, hints at an even larger true effect. This conservative approach can help to prevent overestimation of the treatment effect, providing a more reliable and possibly more realistic lower-bound estimate of the intervention's impact.

Identification results

In this appendix, we show the identification of the causal target parameter, $\Psi(P) = \mathbb{E}_P(Y) - \mathbb{E}_P(Y^d)$. In particular, we show that under the randomization and positivity assumptions,

$$\Psi(P_0) = \mathbb{E}_0(Y) - \mathbb{E}_0(Y^d) = \mathbb{E}_0(Y) - \mathbb{E}_0\mathbb{E}_0(Y \mid A = d(A, W; \delta), W).$$

Under assumptions

- (A1.) randomization: $Y^d \perp A \mid W$,
- (A2.) positivity: if $(a, w) \in (\mathcal{A}, \mathcal{W})$, then $(d(a, w; \delta), w) \in (\mathcal{A}, \mathcal{W})$,
- (A3.) consistency (implied by NPSEM): if $A_i = a$, then $Y_i = Y_i^a$ for all individual i in the population,

we have

$$\begin{aligned}
&\mathbb{E}_0(Y) - \mathbb{E}_0(Y^d) \\
&= \mathbb{E}_0(Y) - \int_{a\in\mathcal{A}}\int_{w\in\mathcal{W}} \mathbb{E}_0(Y^d \mid A = a, W = w) p_{A,0}(a \mid w) p_{W,0}(w) d\nu(a, w) \text{ (by A1.)} \\
&= \mathbb{E}_0(Y) - \int_{a\in\mathcal{A}}\int_{w\in\mathcal{W}} \mathbb{E}_0(Y \mid A = d(a, w; \delta), W = w) p_{A,0}(a \mid w) p_{W,0}(w) d\nu(a, w) \text{ (by A3.)} \\
&= \mathbb{E}_0(Y) - \mathbb{E}_0\mathbb{E}_0(Y \mid A = d(A, W; \delta), W).
\end{aligned}$$

A2. ensures that the conditional expectation, i.e. $\mathbb{E}_0(Y \mid A = d(A, W; \delta), W)$ is well-defined.

Details on TMLE

We provide some justifications of using TMLE and an overview of the TMLE steps. For a more detailed discussion on TMLE for shift intervention target parameters, we refer to chapter 14 of [20].

Our estimand is given by

$$\Psi(P_0) = \mathbb{E}_0(Y) - \mathbb{E}_0(\bar{Q}_0(d(A, W; \delta), W)).$$

We estimate $\Psi^{(1)}(P_0) = \mathbb{E}_0(Y)$ by taking the empirical mean of the observed Y, i.e., the estimator is given by,

$$\hat{\psi}^{(1)} = \frac{1}{n} \sum_{i=1}^{n} Y_i.$$

$\hat{\psi}^{(1)}$ has efficient influence function $D^{(1)}(P_0)(O) = Y - \Psi^{(1)}(P_0)$, and we also have

$$\sqrt{n}(\hat{\psi}^{(1)}(P_n) - \Psi^{(1)}(P_0)) \to^d \text{Normal}(0, \text{Var}(D^{(1)}(P_0)(O))).$$

We use the TMLE framework to estimate $\hat{\psi}^{(2)}$. First, the efficient influence function of $\Psi^{(2)}$ at P_0 is

$$D^{(2)}(P_0)(O) = H(A, W)(Y - \bar{Q}_0(A, W)) + \bar{Q}_0(d(A, W; \delta), W) - \Psi^{(2)}(P_0),$$

where $H(A, W) = \mathbb{1}(A < u(W))g_0(A - \delta \mid W)/g_0(A \mid W) + \mathbb{1}(A \geq U(W) - \delta)$.

TMLE involves the following steps:

1. Estimate g_0 and $\bar{Q}_0$ using super learner, the learner libraries and their hyperparameters are available in Appendix C.

2. For each patient i, evaluate the clever covariate $H_n(A_i, W_i)$ by plugging in super learner estimates g_n and $\bar{Q}_n$ of g_0 and $\bar{Q}_0$.

3. Fit a logistic regression model $\text{logit}Q_n^*(A, W) = \text{logit}\bar{Q}_n(A, W) + \epsilon$, with $H_n(A, W)$ as the weights, to obtain an estimate ϵ_n for ϵ.

4. The TMLE estimator is then $\hat{\psi}_n = 1/n \sum_{i=1}^{n} \bar{Q}_n^*(d(A_i, W_i), W_i)$.

Under regularity conditions stated in chapter 14 of [20],

$$\sqrt{n}(\hat{\psi}^{(2)}(P_n) - \Psi^{(2)}(P_0)) \rightarrow^d \text{Normal}(0, \text{Var}(D^{(2)}(P_0)(O))).$$

By delta method, the efficient influence function for Ψ at P_0 is $D(P_0)(0) = D(P_1)(0) - D(P_2)(0)$, and we estimate the variance as $\hat{\text{Var}}(D(P_n)(O)) = 1/n \sum_{i=1}^{n} D(P_n)(O_i)^2$. Therefore, a Wald-type 95% confidence interval can be constructed as

$$\hat{\psi}(P_n) \pm 1.96\sqrt{\hat{\text{Var}}(D(P_n)(O))/n}.$$

Super learner libraries

TMLE requires estimations of both the outcome regression $\bar{Q}_0(A, W) = \mathbb{E}_0(Y \mid A, W)$ and the conditional density of intervention (a.k.a generalized propensity score) $g_0(A \mid W) = p_{A,0}(A \mid W)$. We provide the learners and their hyperparameters in table 2 and 3.

**Learner**	**Hyperparameters**
Lrnr_mean	Default
Lrnr_glm_fast	Default
Lrnr_xgboost	Default
Lrnr_xgboost	Number of fitting iterations: 1000,<br>maximum depth of a tree: 6,<br>learning rate: 0.1
Lrnr_ranger	Number of trees: 500
Lrnr_glmnet	alpha: 1 (L1-penalized regression),<br>number of folds: 3
Lrnr_glmnet	alpha: 0 (L2-penalized regression),<br>number of folds: 3
Lrnr_glmnet	alpha: 0.5,<br>number of folds: 3
Lrnr_earth	Default
Lrnr_bayesglm	Default
Lrnr_dbarts	Number of posterior draws after burn in: 1000
SL.gam	Default

Table 2: Super learner library for the outcome regression

**Learner**	**Hyperparameters**
Lrnr_density_semiparametric	Mean learner: Lrnr_glm_fast
Lrnr_density_semiparametric	Mean learner: Lrnr_glm_fast, variance learner: Lrnr_glm_fast

Table 3: Super learner library for the generalized propensity score

Code

The code for all analyses can be found on the github repository: https://github.com/tq21/web-appendix-metformin-adherence-covid19

Appendix D: Additional figure

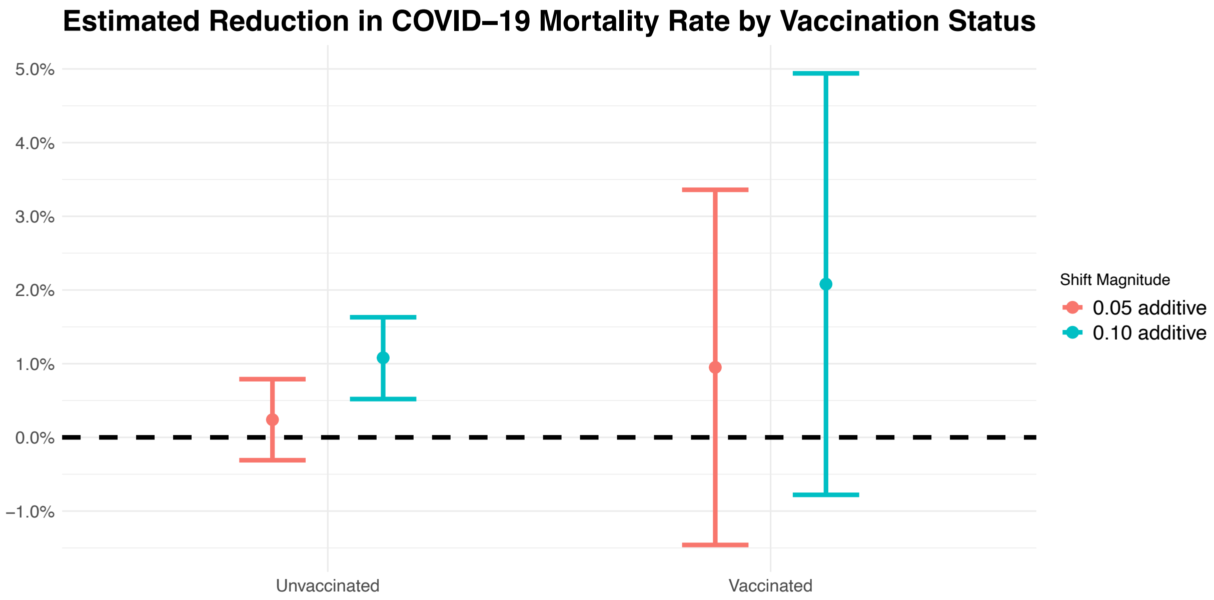

Figure 5: Estimated reduction in COVID-19 post-infection mortality rate by vaccination status under 0.05 and 0.10 additive shifts in metformin adherence, adjusting for confounders. The error bars are 95% confidence intervals obtained using the efficient influence function approach (see Appendix C).
